# Genetic architecture of fatty acid composition in the *longissimusdorsi* muscle revealed by genome-wide association studies on diverse pig populations

**DOI:** 10.1186/s12711-016-0184-2

**Published:** 2016-01-21

**Authors:** Wanchang Zhang, Junjie Zhang, Leilei Cui, Junwu Ma, Congying Chen, Huashui Ai, Xianhua Xie, Lin Li, Shijun Xiao, Lusheng Huang, Jun Ren, Bin Yang

**Affiliations:** State Key Laboratory for Pig Genetic Improvement and Production Technology, Jiangxi Agricultural University, Nanchang, 330045 China

## Abstract

**Background:**

Fatty acid composition in muscle is an important factor that affects the nutritive value and taste of pork. To investigate the genetic architecture of fatty acid composition of pork, we measured fatty acid contents in *longissimus dorsi* muscle of 1244 pigs from three divergent populations and conducted genome-wide association studies (GWAS) for fatty acid contents.

**Results:**

We detected 26 genome-wide significant quantitative trait loci (QTL) on eight chromosomes (SSC for *Sus scrofa*) for eight fatty acids. These loci not only replicated previously reported QTL for C18:0 on SSC14 and C20:0 on SSC16, but also included several novel QTL such as those for C20:1 on SSC7, C14:0 on SSC9, and C14:0, C16:0 and C16:1 on SSC12. Furthermore, we performed a meta-analysis of GWAS on five populations, including the three populations that were investigated in this study and two additional populations that we had previously examined. This enhanced the strength of the associations detected between fatty acid composition and several marker loci, especially for those for C18:0 on SSC14 and C20:0 on SSC16. The genes *ELOVL5*, *ELOVL6*, *ELOVL7*, *FASN*, *SCD* and *THRSP*, which have functions that are directly relevant to fatty acid metabolism, are proximal to the top associated markers at six significant QTL.

**Conclusions:**

The findings improve our understanding of the genetic architecture of fatty acid composition in pork and contribute to further fine-map and characterize genes that influence fatty acid composition.

**Electronic supplementary material:**

The online version of this article (doi:10.1186/s12711-016-0184-2) contains supplementary material, which is available to authorized users.

## Background

Pig meat represents about 40 % of the red meat consumed by humans [1]. Fatty acids are essential cellular components and a major source of energy for animals. Fatty acid composition is closely related to the nutritive value and the taste of meat. Saturated fatty acids such as C14:0 and C16:0 are risk factors for cardiovascular diseases such as coronary heart disease and atherosclerosis in humans [2]. In contrast, unsaturated fatty acids, especially omega-3 fatty acids, are beneficial to human health [3]. Identifying genomic regions associated with fatty acid composition in muscle would enable us to develop molecular breeding technologies to improve pork quality. In humans, fatty acid composition in muscle has been associated with insulin resistance [4]. Since the pig represents an important biomedical model for human diseases [5], dissection of the genetic architecture of fatty acid composition in pork can also provide insights into the molecular mechanisms that underlie fatty acid metabolism in humans.

Genome-wide association studies (GWAS) have been performed to identify genomic regions associated with a variety of traits in pigs [6–8] and significant quantitative trait loci (QTL) that influence fatty acid composition have been identified in several pig populations. These QTL harbor strong candidate genes such as *SCD* [9], *ELOVL6* [10] and *ELOVL7* [9]. However, the genetic basis that underlies variation in fatty acid composition is still largely unclear. For instance, further investigations are needed to reveal additional QTL that affect fatty acid composition in pork from diverse breeds. Moreover, a meta-analysis of results from multiple populations is required to improve the detection power of GWAS for fatty acid composition in pork.

In our previous study, we reported GWAS on fatty acid composition in *longissimus dorsi* muscle samples from a White Duroc × Erhualian F_2_ intercross (hereafter referred to as F_2_, n = 591) and Sutai pigs (n = 282) [9]. Here, we recorded fatty acid contents in the *longissimus dorsi* muscle and obtained ~60 K single nucleotide polymorphism (SNP) genotypes on 1244 pigs from three additional populations: two Chinese indigenous breeds (Laiwu, n = 305; Erhualian, n = 331) and a Duroc × (Landrace × Yorksire) (DLY) three-way hybrid commercial population (n = 608). We performed GWAS on each of these three populations and identified a list of novel significant QTL that harbor interesting candidate genes. We also conducted a meta-analysis of GWAS on five populations, including the three populations (Laiwu, Erhualian and DLY) that we tested in this study and the two populations (F_2_ and Sutai) that we previously investigated. The large sample size and broad genetic background of our tested populations allowed us not only to confirm previously reported QTL, but also to identify novel QTL and candidate genes.

## Methods

### Animals

Five pig populations were used in this study, including Laiwu, Erhualian, DLY, F_2_, and Sutai pigs. Laiwu is a Chinese indigenous pig breed that was originally distributed in Laiwu, Shangdong province. This breed is known for its unusually high intramuscular fat content (9–12 %) [11]. Erhualian pigs are mainly located in Wuxi, Jiangsu Province, and are famous for their large litter size [11]. We purchased 385 Laiwu and 390 Erhualian piglets at the age of 90 days from nucleus farms of these two breeds in Shandong and Jiangsu provinces, respectively. These pigs were selected to represent offspring of all boars and the majority of sows in these two farms. The Laiwu pigs were derived from 25 sires and 115 dams and the Erhualian pigs were offspring of 11 sires and 55 dams. These animals were transferred to and raised in a pig farm in Nanchang, Jiangxi Province. Boars were castrated before day 90. All Erhualian and Laiwu pigs were fed a corn-soybean based diet containing 16 % crude protein, 3100 kJ digestible energy and 0.78 % lysine under standard management conditions. A total of 333 Laiwu and 336 Erhualian pigs were uniformly slaughtered at 300 ± 3 days in 18 and 11 batches, respectively. A total of 698 DLY pigs were purchased from a commercial pig farm in Xiushui, Jiangxi Province. The DLY boars were castrated at day 25 and all pigs were fed a corn-soybean diet containing 16 % crude protein, 3132 kJ digestible energy and 0.85 % lysine, and were slaughtered at 180 ± 3 days in 22 batches.

To conduct a meta-analysis of GWAS, we also used the 60 K SNP genotypes and fatty acid composition phenotypes from two other populations: F_2_ and Sutai pigs. The pedigree, management and genotype information of these two populations were described previously [9]. In brief, the F_2_ population comprised 1912 F_2_ pigs that were derived from two White Duroc founder boars and 17 Chinese Erhualian founder sows. The Sutai population is a Chinese synthetic pig line that originated from a cross between Chinese Taihu and European Duroc pigs and then artificially selected for more than 18 generations [11]. Both F_2_ and Sutai pigs were fed with the same diet that was used for the Erhualian and Laiwu pigs during the fattening period.

### Ethics statements

All the experiments that involved animals were carried out in accordance with the approved guidelines by the Ministry of Agriculture of China. Approval was obtained from the ethics committee of Jiangxi Agricultural University before the experiment.

### Phenotype recording

Approximately 50 g of *longissimus dorsi* tissue was dissected from the 3rd to 4th lumbar vertebrae region of each pig and frozen in liquid nitrogen within 30 min post-mortem, and then stored at −80 °C for further use. About 10 g of *longissimus dorsi* tissue was ground and then treated with a 3:1 chloroform–methanol solution according to [12]. Then, 2 mg of extracted lipids was re-dissolved in 2 mL of n-hexane and 1 mL of KOH (0.4 M) for saponification and methylation. Fatty acid methyl esters were processed with a GC2010 Plus Gas Chromatograph (Shimadzu), following manufacturer recommendations. Signals for each fatty acid were quantified relative to standard reference reagents (Sigma-Aldrich) and the percentage of each fatty acid relative to total fatty acids was used as phenotype for further analyses. The same measurement procedures were used for all five populations.

### Genotype data

A standard phenol/chloroform method was used to extract genomic DNA from ear tissue of each pig. All animals were genotyped with Illumina Porcine SNP60 BeadChips (v2) [13] according to the manufacturer’s protocol. We used the same quality control procedures on genotype data for all tested populations. Briefly, we selected SNPs that had a call rate greater than 0.9 and a minor allele frequency (MAF) greater than 0.01, and individuals that had a genotype call rate higher than 0.9, for further analyses. All quality control procedures were implemented by Plink v1.07 [14].

### Estimation of phenotypic correlations

To estimate phenotypic correlations between different fatty acid contents, we adjusted the content of each fatty acid in each population separately by treating sex and slaughter batch as fixed effects and polygenic effects as random effects in a single-trait linear mixed model implemented by the *polygenic* function in the R package GenABEL [15]. The variance–covariance matrix of polygenic effects was estimated based on whole-genome SNP genotype data. Phenotypic correlations were measured as spearman correlation coefficients. Heatmaps of the correlation matrix were plotted by the “heatmap.2” function of the gplots package in R software (https://cran.r-project.org/web/packages/gplots/).

### Estimation of heritability and GWAS

The proportion of phenotypic variance explained by whole-genome SNP genotypes i.e., heritability, was estimated using the −l mm procedure of GEMMA [16] based on the genomic relationship matrix. Sex and batch were included as fixed effects and the estimation was done separately for each population. For GWAS, we assessed the association between phenotypes and each SNP across the genome under the following linear mixed model:

$${\textbf{y = Xb + Za + kg + e}},$$

where y is a vector of phenotypic values, **X**, **Z**, and **k **are incidence matrices for fixed effects (sex and batch), polygenic effects and SNP genotypes, respectively. **Z** is an identity matrix, **k** is a vector of 0, 1, 2 values, where 0 and 2 represent the two alternative homozygotes and 1 denotes the heterozygote at the evaluated SNP,** b** is a vector of fixed effects, **a** is a vector of polygenic effects with distribution: N (0,**G***σ*_*A*_^2^), with **G** the genomic relationship matrix based on genome-wide SNPs and *σ*_*A*_^2^ the additive genetic variance, g is the additive genetic effect of the tested SNP, and **e** is a vector of residuals. We identified significant SNPs through a stepwise forward selection in multiple rounds of genome scans, where the most significant SNPs that were identified in previous genomic scans were included as additional fixed effects in the model for the next round of scan until no SNP passed the suggestive significant threshold [17]. The threshold for defining a suggestive significant SNP was set to 1/N_snp_, where N_snp_ was the number of SNPs tested in genome-wide scans. Genome-wide significance thresholds were set to 0.05/N_snp_.

The GWAS meta-analysis was performed using the METAL program [18], which tests the combined effects and standard errors of effects of each common SNP across the tested populations by taking sample size and direction of genotype effects into account. The phenotypic variance explained by significant GWAS SNPs was estimated as (V_reduce−_V_full_)/V_reduce_, where V_reduce_ and V_full_ are the residual variances of ordinary linear models with and without including SNP genotypes as predictor variables, respectively.

### Characterization of candidate genes

To identify functionally plausible candidate genes near the genome-wide significant SNPs, we examined annotated genes around the region that was centered at each top SNP that was identified by GWAS in the *Sus scrofa* assembly (Build 10.2). Because annotation of the pig reference genome is far from perfect, we also investigated homologous segments in other mammalian species, including mouse, cattle and humans (http://genome.ucsc.edu). Among the multiple genes that were found within target regions, we prioritized candidate genes according to their physical distance to the top SNPs and their functional relevance to fatty acid metabolism.

## Results and discussion

### Phenotypes

**Fig. 1:**
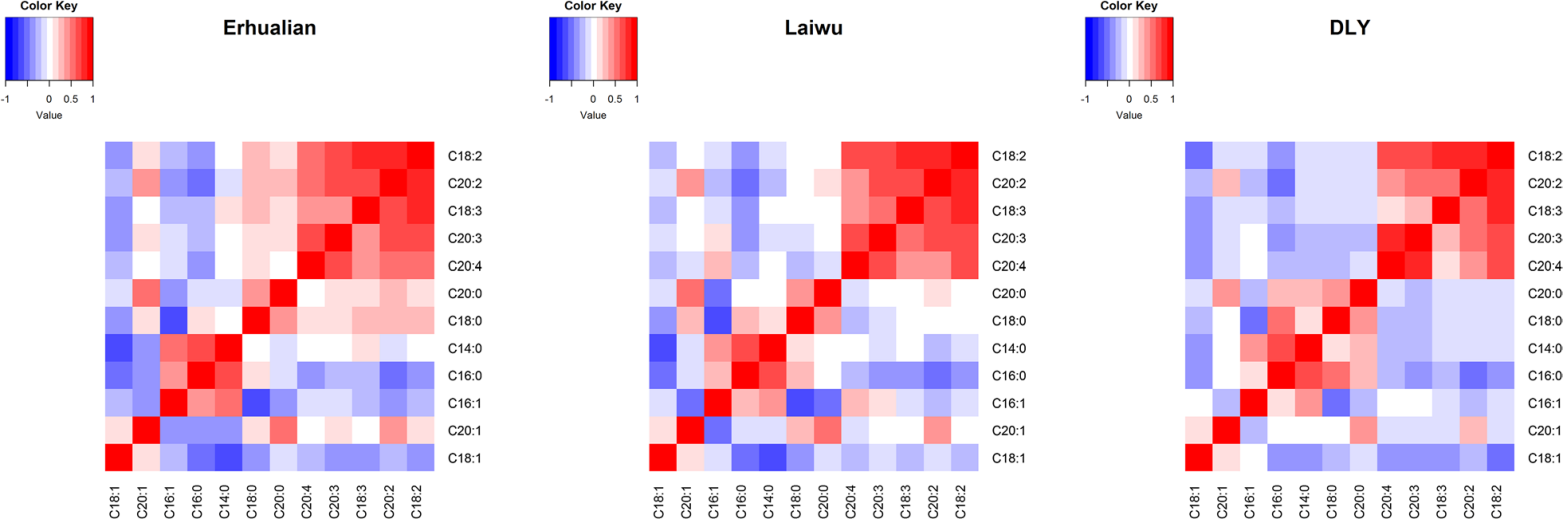
Heatmap of phenotypic correlations between 12 major fatty acids in three populations

We focused on 12 fatty acids that account for ~95 % of the total fatty acids (Table 1). The most abundant fatty acid was C18:1, followed by C16:0 and C18:0, across the populations analyzed. Heritability estimates for most fatty acids ranged from 0.4 to 0.8 (Table 1), which suggests that the contribution of genetic effects to phenotypic variation in fatty acid composition is considerable. We assessed phenotypic correlations between fatty acid contents for the Laiwu, Erhualian and DLY populations. Briefly, polyunsaturated fatty acids, including C18:2, C18:3, C20:2, C20:3 and C20:4, were positively correlated with each other. These fatty acids were clustered into a module that was distinct from the saturated and mono-unsaturated fatty acids (Fig. 1). Saturated fatty acids (C14:0, C16:0, C18:0 and C20:0) were also positively correlated with each other. These correlation patterns were conserved across the three populations (Fig. 1).

**Table 1:** Summary statistics for fatty acid composition in three pig populations

Trait	Laiwu			Erhualian			DLY		
	N	Mean ± SD	*h* ^*2*^ ± SE	N	Mean ± SD	*h* ^*2*^ ± SE	N	Mean ± SD	*h* ^*2*^ ± SE
Myristic (C14:0)	305	1.34 ± 0.12	0.67 ± 0.09	331	1.34 ± 0.18	0.81 ± 0.08	608	1.31 ± 0.12	0.51 ± 0.08
Palmitic (C16:0)	305	26.50 ± 1.00	0.56 ± 0.08	331	25.00 ± 1.30	0.73 ± 0.08	608	23.88 ± 1.22	0.56 ± 0.08
Palmitoleic (C16:1*n*-7)	305	3.87 ± 0.60	0.70 ± 0.06	331	3.62 ± 0.68	0.77 ± 0.08	608	3.91 ± 0.46	0.67 ± 0.09
Stearic (C18:0)	305	12.09 ± 1.11	0.57 ± 0.09	331	12.58 ± 1.25	0.75 ± 0.09	608	11.36 ± 1.14	0.64 ± 0.09
Oleic (C18:1*n*-9)	305	46.42 ± 1.58	0.48 ± 0.10	331	46.17 ± 2.26	0.76 ± 0.09	608	45.36 ± 2.04	0.46 ± 0.09
Linoleic (C18:2*n*-6)	305	3.63 ± 0.80	0.01 ± 0.05	331	4.46 ± 0.89	0.48 ± 0.10	608	5.40 ± 1.51	0.29 ± 0.09
Linolenic (C18:3*n*-3)	305	0.11 ± 0.03	0.12 ± 0.09	331	0.13 ± 0.03	0.45 ± 0.10	608	0.19 ± 0.07	0.26 ± 0.09
Arachidic (C20:0)	305	0.19 ± 0.03	0.72 ± 0.11	331	0.25 ± 0.05	0.73 ± 0.08	608	0.19 ± 0.03	0.77 ± 0.08
Eicosenoic (C20:1*n*-9)	305	0.75 ± 0.15	0.82 ± 0.07	331	0.74 ± 0.13	0.63 ± 0.10	608	0.86 ± 0.11	0.46 ± 0.08
Eicosadienoic (C20:2*n*-6)	305	0.20 ± 0.04	0.18 ± 0.10	331	0.25 ± 0.05	0.59 ± 0.09	608	0.28 ± 0.07	0.43 ± 0.09
Homolonolenic (C20:3*n*-6)	305	0.05 ± 0.01	0.26 ± 0.10	331	0.08 ± 0.02	0.39 ± 0.10	602	0.09 ± 0.03	0.18 ± 0.08
Arachidonic (C20:4*n*-6)	305	0.18 ± 0.08	0.20 ± 0.10	331	0.25 ± 0.09	0.46 ± 0.10	608	0.48 ± 0.29	0.13 ± 0.07

N stands for the number of individuals in each population

*h* ^2^ represents the heritability of each trait for each population; heritabilities were estimated using the −l mm procedure of GEMMA based on genome-wide SNP genotypes [16]

### GWAS in the Erhualian, Laiwu and DLY populations

After quality control, 35,974 SNPs for 305 Erhualian pigs, 49,343 SNPs for 331 Laiwu pigs, and 56,216 SNPs for 608 DLY pigs were retained for GWAS. We discarded 24,893, 11,249 and 4806 SNPs with a MAF lower than 0.01 for the Erhualian, Laiwu and DLY populations, respectively. Different numbers of rare SNPs significantly resulted in different numbers of qualified SNPs in the three populations. This was as expected because SNPs on the porcine Illumina 60 K chips were selected mainly based on European pigs that have diverged from Chinese indigenous pigs [13]. Therefore, ascertainment bias was unavoidable, leading to a higher proportion of rare SNPs for the Chinese Laiwu and Erhualian pigs than for the DLY pigs.

**Fig. 2:**
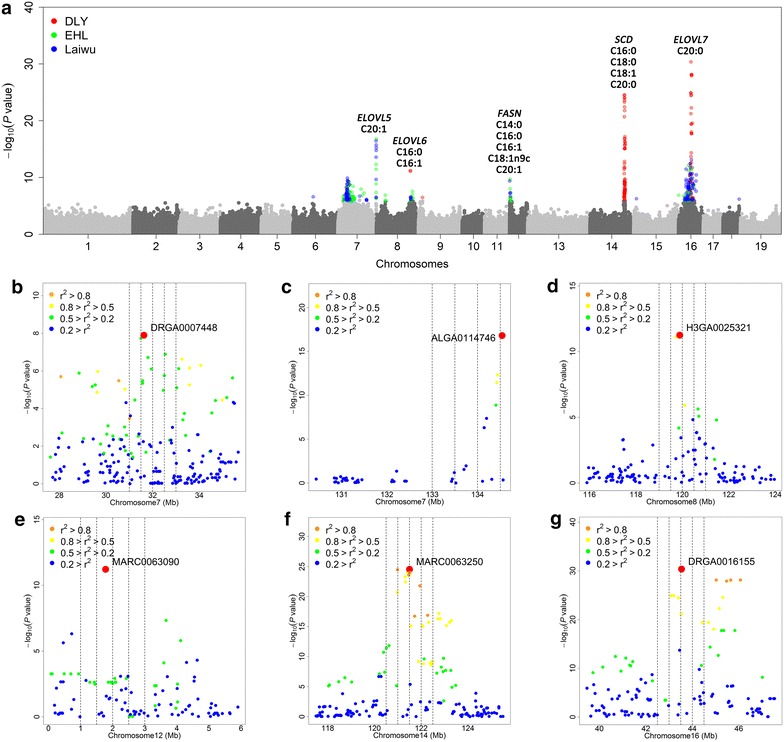
Significant GWAS SNPs and candidate genes for fatty acid composition in Erhualian, Laiwu and DLY pigs. **a** SNPs that surpass the genome-wide significant threshold are denoted with different colors in each population (*red* for DLY, *green* for Erhualian, and *blue* for Laiwu). Candidate genes at several genome-wide significant loci are indicated. Panels **b**–**g** represent the significant regional plots for the SNP that affects the C20:1 content on SSC7 in Laiwu pigs (**b**), the C20:1 content on SSC7 in Erhualian pigs (**c**), the C16:1 content on SSC8 in DLY pigs (**d**), the C14:0 content on SSC12 in Erhualian pigs (**e**), the C18:0 content on SSC14 in DLY pigs (**f**) and the C20:0 content on SSC16 in DLY pigs (**g**), respectively. The top SNP in each plot is indicated in red and the surrounding SNPs in different colors represent different linkage disequilibrium extents (*r* ^2^) to the top SNP

We identified 26 genome-wide significant QTL on eight chromosomes for eight fatty acid contents (Fig. 2a; Table 2). These QTL explained from 3.3 to 35.2 % of the phenotypic variance for a fatty acid phenotype (Table 2). The QTL for C18:0 content on SSC14 and C20:0 content on SSC16 replicated QTL that were previously reported for these traits in the F_2_ and Sutai populations [9], and the QTL for C16:1 content on SSC8 overlapped with the QTL for this trait identified in an Iberian × Landrace intercross [7]. To our knowledge, the other QTL were detected for the first time, including the QTL for C20:1 around 134.54 Mb on SSC7, C14:0 around 13.83 Mb on SSC9, and C14:0 and C16:0 around 1.7 Mb on SSC12 (Table 2).

**Table 2:** Genome-wide significant QTL for fatty acid composition in Erhualian, Laiwu and DLY pig populations

Chr	Trait	Pop	Nsnp	Range of Nsnp (Mb)	Top SNP	Position (bp)	*P*-value	Var (%)	Candidates
6	C16:1	Laiwu	1	71.01	ASGA0106005	71011913	1.97E−07	4.95	
7	C20:1	Laiwu	18	27.35–31.80	H3GA0020505	29416373	2.25E−08	7.91	
7	C14:0	Laiwu	15	29.54–33.59	ALGA0039950	31500144	2.60E−08	17.78	
7	C16:1	Laiwu	21	29.42–34.06	DRGA0007448	31628039	1.27E−08	8.03	
7	C20:2n6	Laiwu	6	29.42–34.06	DRGA0007448	31628039	5.61E−07	6.55	
7	C20:1	Laiwu	9	133.96–134.54	ASGA0037322	133962789	3.54E−17	35.18	*ELOVL5*
7	C20:1	Erhualian	6	134.15–134.54	ALGA0114746	134540651	1.51E−17	26.43	
8	C16:1	DLY	7	119.73–121.47	H3GA0025321	119887525	1.54E−13	8.31	*ELOVL6*
8	C16:0	Laiwu	20	118.82–123.41	ALGA0049254	120996107	2.66E−07	8.89	
8	C16:1	Erhualian	11	125.08–129.51	ALGA0049349	127072110	6.18E−09	9.77	
9	C20:1	DLY	4	6.29–7.58	ALGA0105252	6304559	4.27E−07	3.99	
9	C14:0	DLY	4	13.26–14.31	ALGA0107040	13826057	2.15E−08	3.33	*THRSP*
9	C14:0	DLY	4	13.26–14.31	MARC0100725	14217867	3.23E−07	4.00	
12	C14:0	Laiwu	6	0.25–2.52	ASGA0099260	248014	4.06E−10	9.01	
12	C14:0	Erhualian	2	0.46–4.12	MARC0063090	1779278	6.05E−12	26.81	*FASN*
12	C16:0	Erhualian	3	0.46–4.12	MARC0063090	1779278	9.24E−09	19.56	
12	C16:1	Erhualian	4	0.46–4.12	MARC0063090	1779278	2.01E −10	13.07	
12	C18:1n9c	Erhualian	4	0.46–4.12	MARC0063090	1779278	1.14E−06	17.44	
12	C20:1	Erhualian	5	0.46–4.12	MARC0063090	1779278	1.03E−07	10.97	
14	C16:0	DLY	11	120.39–121.95	ALGA0081087	120972588	4.00E−08	6.25	*SCD*
14	C20:0	DLY	4	120.97–121.50	CASI0010164	121305916	1.04E−07	3.51	
14	C18:0	DLY	45	119.03–123.48	MARC0063250	121500518	2.82E−25	26.35	
14	C16:1	DLY	35	120.51–123.29	ASGA0066120	121515129	1.47E−13	11.13	
16	C20:0	Erhualian	20	32.43–36.85	ASGA0072949	34715842	6.97E−13	12.07	
16	C20:0	DLY	37	41.14–45.83	DRGA0016155	43534471	4.32E−31	31.79	*ELOVL7*
16	C20:0	Laiwu	4	43.53–45.31	DRGA0016169	45313348	7.41E−14	25.50	

*P*-values were calculated using the GenABEL package in R

Phenotypic variation explained by the top SNPs was estimated by (V_reduce_−V_full_)/V_resuce_, where V_reduce_ and V_full_ are the residual variances of models with and without including SNP genotypes as predictor variables, respectively

*Chr* chromosome, *Pop* population, *N*_*snp*_ number of significant SNPs, *Position* genomic position on the Sscrofa 10.2 pig genome assembly, *Var* (*%*) percentage of phenotypic variance explained by each locus

### Shared and specific QTL across populations

We also conducted a meta-analysis of GWAS to assess the combined association signals for the five populations: F_2_, Sutai, Erhualian, Laiwu and DLY. The meta-analysis did not identify new QTL but enhanced the strength of associations between fatty acid composition and several markers, especially for the QTL for C18:0 on SSC14 and C20:0 on SSC16 (see Additional file 1: Figure S1). The *P* value of the top SNP (CASI0010164) for C18:0 increased from 2.82 × 10^−25^ for DLY pigs to 5.06 × 10^−44^ in the meta-analysis, and the *P*-value of the top SNP (DRGA0016155) for C20:0 on SSC16 increased from 4.32 × 10^−31^ in DLY pigs to 3.85 × 10^−47^ in the meta-analysis. The direction of SNP effects was concordant across the five populations, with allele *C* at SNP CASI0010164 and allele *G* at SNP DRGA0016155 increasing C18:0 and C20:0 contents, respectively. This indicates that common or closely-linked functional variants may be responsible for the GWAS signals in these populations.

We identified several genomic regions that showed genome-wide significant association signals in multiple populations, including the regions between 134.0 and 134.5 Mb on SSC7 for C20:1, between 120.0 and 127.1 Mb on SSC8 for C16:1, and between 0.21 and 1.87 Mb on SSC12 for C14:0 (Table 2). The extent of linkage disequilibrium (LD) is usually small in diverse populations. For example, LD at *r*^*2*^ = 0.3 extends across 10.5 and 125 kb in Chinese and European pigs, respectively [19]. The 60 K SNPs on the Illumina chip are not likely the causative mutations for the observed associations. However, it is reasonable to hypothesize that the population-shared signals observed here arise from common causative mutations. The top SNPs for multiple associations across diverse populations must be in the vicinity of the causative mutations, providing insights to further characterize these causative mutations.

### Candidate genes for genome-wide significant QTL

In most mammals, fatty acids up to palmitate (C16:0) are synthesized de novo from malonyl-CoA and acetyl-CoA by fatty acid synthetases (FAS) in the liver and fat tissues [20]. However, some fatty acids (essential fatty acids), such as linoleic acids (C18:2*n*-6) and linolenic acids (C18:3*n*-3), are normally found in vegetables and nuts and cannot be de novo synthesized in mammals. Within cells, fatty acids can be metabolized or transformed by different elongases and desaturases. For example, SCD (stearoyl-CoA desaturase) desaturates the saturated fatty acids C16:0 and C18:0 to the mono-unsaturated fatty acids C16:1*n*-7 and C18:1*n*-9. Different elongases (ELOVL2 and ELOVL5) and desaturases (Δ5 and Δ6 desaturases) are involved in catalyzing *n*-3 and *n*-6 polyunsaturated fatty acids [20]. Therefore, it is conceivable that altered enzymatic activities of these synthetases, elongases and desaturases affect fatty acid composition in cells or tissues. This contributes fundamental knowledge to characterize candidate genes for genome-wide significant QTL.

On SSC7, we detected two significantly associated regions in the genome of the Laiwu and Erhualian pigs but not of DLY pigs (Table 2; Fig. 2). The region around 31.63 Mb was associated with C14:0 and C16:1 contents in Laiwu pigs (Fig. 2b). The other region, around 134.54 Mb, was associated with the C20:1 content in both Erhualian and Laiwu pigs. The top SNP ALGA0114746 for the 134.54 Mb region in the Erhualian pigs is located at about 5 kb upstream of the *ELOVL5* (*elongation of very long chain fatty acid protein 5)* gene (between 134.54 and 134.62 Mb) (Fig. 2c). *ELOVL5* encodes an elongase that plays an important role in determining de novo synthesis of monounsaturated fatty acid in mice [21]. Therefore, *ELOVL5* is a strong candidate for this QTL that affects C20:1 content.

On SSC8, we found significant signals for C16:0 and C16:1 contents in Erhualian, Laiwu and DLY pigs (Table 2). The most significantly associated SNP (*P* = 1.54 × 10^−13^) was H3GA0025321 at 119.88 Mb for C16:1 for DLY pigs (Fig. 2d). The top SNP (ALGA0049254) for C16:0 for Laiwu pigs was at 121.00 Mb. This region (between 119.88 and 121.00 Mb) overlapped with the QTL (between 117.82 and 120.10 Mb) for C16:1 that was identified for Iberian × Landrace F_2_ and backcross populations [7, 22]. This region contains the *ELOVL6* (*elongation of very long chain fatty acid protein 6*) gene at 120.12 Mb. *ELOVL6* plays a key role in catalyzing elongation of 16–18 carbon fatty acids [23]. The level of expression of this gene has been associated with C16:0 and C16:1 contents in pig muscle [10]. Interestingly, allele *C* at the top SNP (H3GA0025321) was associated with decreased C16:1 content and increased C18:1 content (*P* = 8.9 × 10^−5^), which is consistent with the enzymatic activity of *ELOVL6*. Similar scenarios were also found for the Erhualian and Laiwu pigs.

At 13.82 and 14.21 Mb on SSC9, we found SNPs that were significantly associated with C14:0 content in DLY pigs but not in Erhualian or Laiwu pigs (Table 2). The top SNP at 13.82 Mb was located in an intron of the *NDUFC2* (*NADH dehydrogenase 1 subunit C2*) gene, which has no apparent functional relevance to fatty acid metabolism. We further investigated homologous regions in other species, including humans and mice, and found that the region that is homologous to the segment between 13.81 and 13.82 Mb on SSC9 contains the *THRSP* (*thyroid hormone responsive*) gene. *THRSP* is involved in the regulation of genes that are involved in fatty acid synthesis [24]. Interestingly, the homologous region in the bovine genome is associated with muscle fatty acid composition in cattle [25]. Therefore, we consider *THRSP* as an interesting candidate gene that warrants further investigation.

On SSC12, we identified significant SNPs for C14:0 content in the region between 0.21 and 1.87 Mb in both Erhualian and Laiwu pigs (Table 2). The top SNP (ASGA0099260, *P* = 4.06 × 10^−10^) detected for the Laiwu pigs was located at 0.25 Mb, which is about 1.52 Mb away from the top SNP (MARC0063090, *P* = 6.05 × 10^−12^) at 1.77 Mb that was identified for Erhualian pigs (Fig. 2e). Allele *A* at the top SNP for Erhualian pigs was significantly associated with increased C14:0, C16:0 and C16:1 contents but with decreased C18:1 and C20:1 contents (Table 2). The same association pattern was found at this QTL for Laiwu pigs, although *P* values of the top SNPs for C16:0 (*P* = 4.3 × 10^−4^), C16:1 (*P* = 1.7 × 10^−4^), C18:1 (*P* = 4.7 × 10^−5^) and C20:1 (*P* = 2.6 × 10^−4^) did not reach the genome-wide significance threshold. For DLY pigs, although ASGA0099260 (MAF = 0.23) and MARC0063090 (MAF = 0.42) had high MAF, no significant association was found. This indicates that causal variant(s) at this QTL may have a population-specific effect in Erhualian and Laiwu pigs. Another possible reason is that there is no genetic variation at the causal variant in DLY pigs. At 1.02 Mb on this chromosome, we found the *FASN* (*fatty acid synthase*) gene, which encodes an enzyme involved in the synthesis of C16:0 from acetyl-CoA and malonyl-CoA [26].

On SSC14, SNPs within a ~500 kb region (between 120.97 and 121.50 Mb) were significantly associated with C16:0, C16:1, C18:0 and C20:0 contents in DLY but not in Erhualian and Laiwu pigs (Table 2). Within this region, the most significant association was observed between MARC0063250 at 121.50 Mb and C18:0 content in DLY pigs (*P* = 2.82 × 10^−25^) (Fig. 2f). The top SNP explained 26.4 % of phenotypic variance. It should be noted that this region overlapped with a previously reported QTL for C18:0 in the F_2_ and Sutai populations [9]. The *SCD* gene at 120.96 Mb was proposed as a strong candidate gene for this QTL. *SCD* encodes a rate-limiting enzyme that preferably catalyzes palmitoyl—(C16:0) and stearoyl—(C18:0) to palmitoleoyl-CoA (C16:1) and oleoyl-CoA (C18:1) [27]. *SCD* polymorphisms have been associated with fatty acid composition in pig, cattle and sheep [28–30]. Interestingly, in this study, allele *A* at the top SNP (MARC0063250) showed positive effects on C16:0 and C18:0 contents but negative effects on C16:1 and C18:1 contents (Table 2), which agrees with the enzymatic activity of SCD.

It should be noted that we identified a significant QTL for C20:0 around 43.51 Mb on SSC16 in Erhualian, Laiwu and DLY pigs, despite the divergent genetic background between Chinese indigenous and DLY pigs. This region also showed significant association signals for C20:0 in the F_2_ and Sutai pigs. In this study, the most significant associated SNP (DRGA0016155, *P* = 4.32 × 10^−31^) was identified for DLY pigs at 43.51 Mb on this chromosome. This SNP was also the top SNP (*P* = 3.85 × 10^−47^) in the meta-analysis. However, the top SNPs differed between the five populations, with distances of 1 to 10 Mb between each other. One explanation is that the marker density of the current 60 K SNP array is still not sufficient to capture the across-population LD between causative variants and SNPs. Another explanation could be that the causative variants differ between populations. In our previous study, we proposed *ELOVL7* (e*longation of very long chain fatty acid protein 7*) at 42.50 Mb as an interesting candidate gene for the SSC16 locus, since *ELOVL7* encodes an elongase that is directly involved in the metabolism of C20:0 and is about 200 kb away from the strongest SNP that was found for Sutai pigs. We note that *ELOVL7* is ~1 Mb away from the top SNP (DRGA0016155) identified for DLY pigs (Fig. 2g) [9].

### Suggestive QTL

**Fig. 3:**
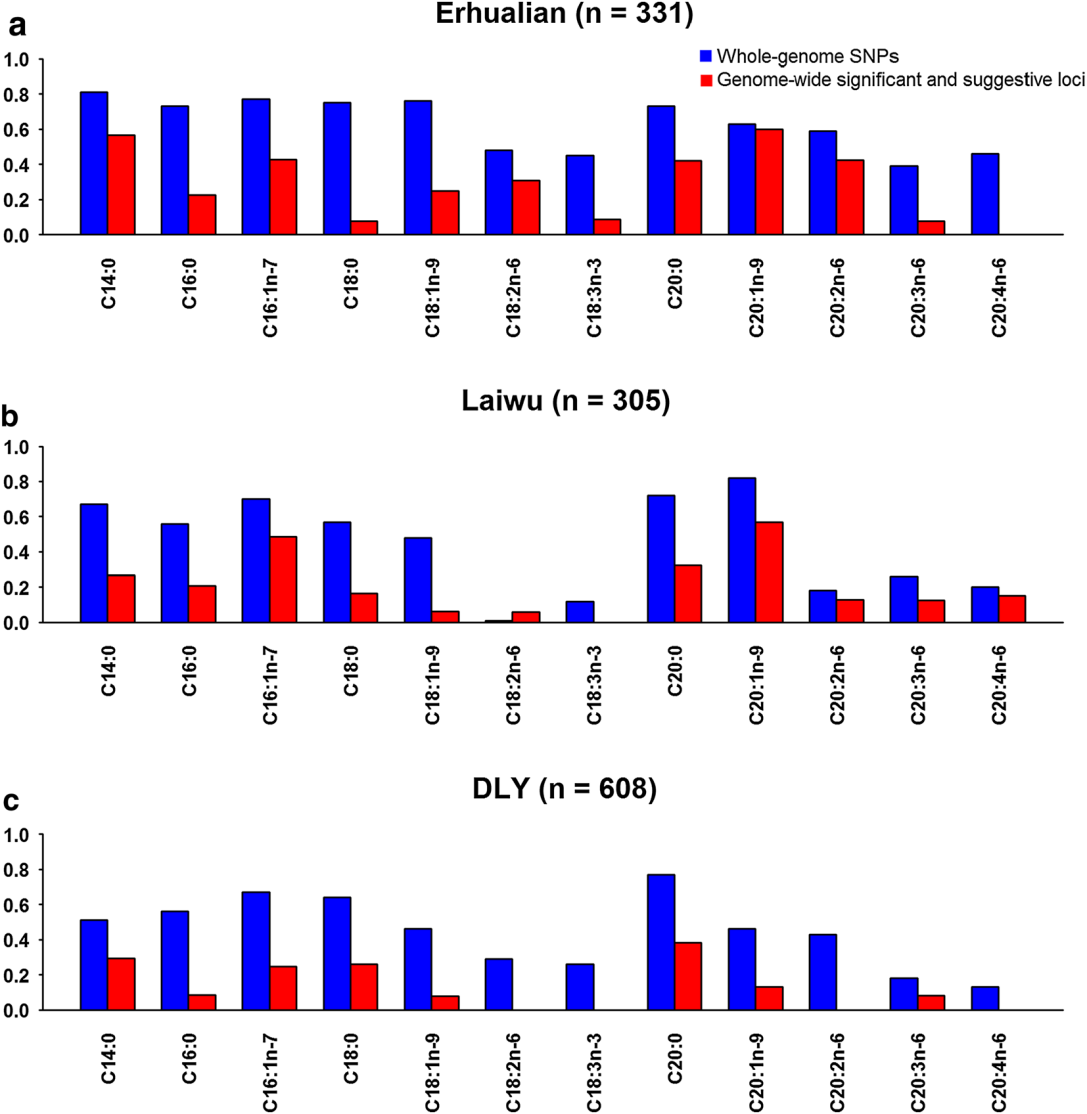
Comparison of the proportions of phenotypic variance explained by both suggestive and genome-wide significant SNPs with those explained by whole-genome SNPs. **a** Erhualian pigs. **b** Laiwu pigs. **c** Duroc × (Landrace × Yorksire) hybrid pigs

At the less strict thresholds for suggestive significance (Erhualian: 1/35974; Laiwu: 1/49343; and DLY: 1/56216), we identified an additional 76 QTL for the contents of 12 fatty acids in Erhualian, Laiwu and DLY pigs (see Additional file 2: Table S1). On average, two suggestive QTL were detected for each fatty acid in each population. Dissecting the molecular basis of these QTL with small effects is still a big challenge. However, these QTL regions harbor markers that can be potentially used in breeding schemes to improve fatty acid composition of meat. We compared the percentage of phenotypic variance explained by all the QTL that surpassed the suggestive significance threshold with the percentage of phenotypic variance that is collectively explained by all SNPs i.e., the marker-based heritability (Fig. 3). We found that the identified QTL explained more than 30 % of the heritability or 20 % of the phenotypic variance for most fatty acids, including C14:0, C16:0, C16:1, C18:0, C18:1, C18:2, C20:0, C20:1 and C20:2, in at least one population. For instance, we identified seven suggestive QTL for C14:0 for Erhualian pigs. These QTL together explained 69.8 % of the heritability and 56.5 % of the phenotypic variance (Additional file 2: Table S1 and Fig. 3). These results indicated that a small number of QTL can account for a considerable proportion of the breeding values for most fatty acid contents, which suggests that genetic improvement for fatty acid composition and pork quality by marker-assisted selection using a small set of SNPs can be considered.

## Conclusions

We investigated fatty acid composition in the *longissimus dorsi* muscle of 1244 pigs from three divergent populations. The correlation patterns of fatty acids revealed that the metabolic pathway of polyunsaturated fatty acids is likely different from those of saturated and mono-unsaturated fatty acids. Through GWAS and GWAS meta-analysis, we detected 26 genome-wide significant QTL on eight chromosomes for eight fatty acids. These QTL not only replicated previously reported QTL but also included several novel QTL, which displayed complex patterns of pleiotropic effects, as well as population-shared and population-specific effects. At six significant QTL, we highlighted candidate genes related to fatty acid metabolism near the top associated SNPs. We showed that a small number of SNPs can explain a considerable proportion of phenotypic variance in the content of most fatty acids for at least one population. This study advances our understanding of the genetic architecture of fatty acid composition in porcine muscle.

## Additional files

10.1186/s12711-016-0184-2 Manhattan plot for the GWAS meta-analysis for the content of 12 fatty acids in five populations, including Erhualian, Laiwu, DLY, Sutai and White Duroc × Erhualian F_2_ intercross. The solid line represents the genome-wide threshold (0.05/28025). The dashed line indicates the suggestive threshold (1/28025). Significant associations that surpass the genome-wide threshold are plotted in different colors on each chromosome. The fatty acid traits associated with the genome-wide significant loci are marked above the top SNPs.
10.1186/s12711-016-0184-2 Loci of suggestive significance identified by GWAS for fatty acid composition in Laiwu, Erhualian and DLY pig populations.
