# Supplementary figures and images for: Genetic architecture of fatty acid composition in the longissimusdorsi muscle revealed by genome-wide association studies on diverse pig populations
Source: Genet Sel Evol. 2016 Jan 21;48:5. doi: 10.1186/s12711-016-0184-2 (PMC4722735; doi:10.1186/s12711-016-0184-2)

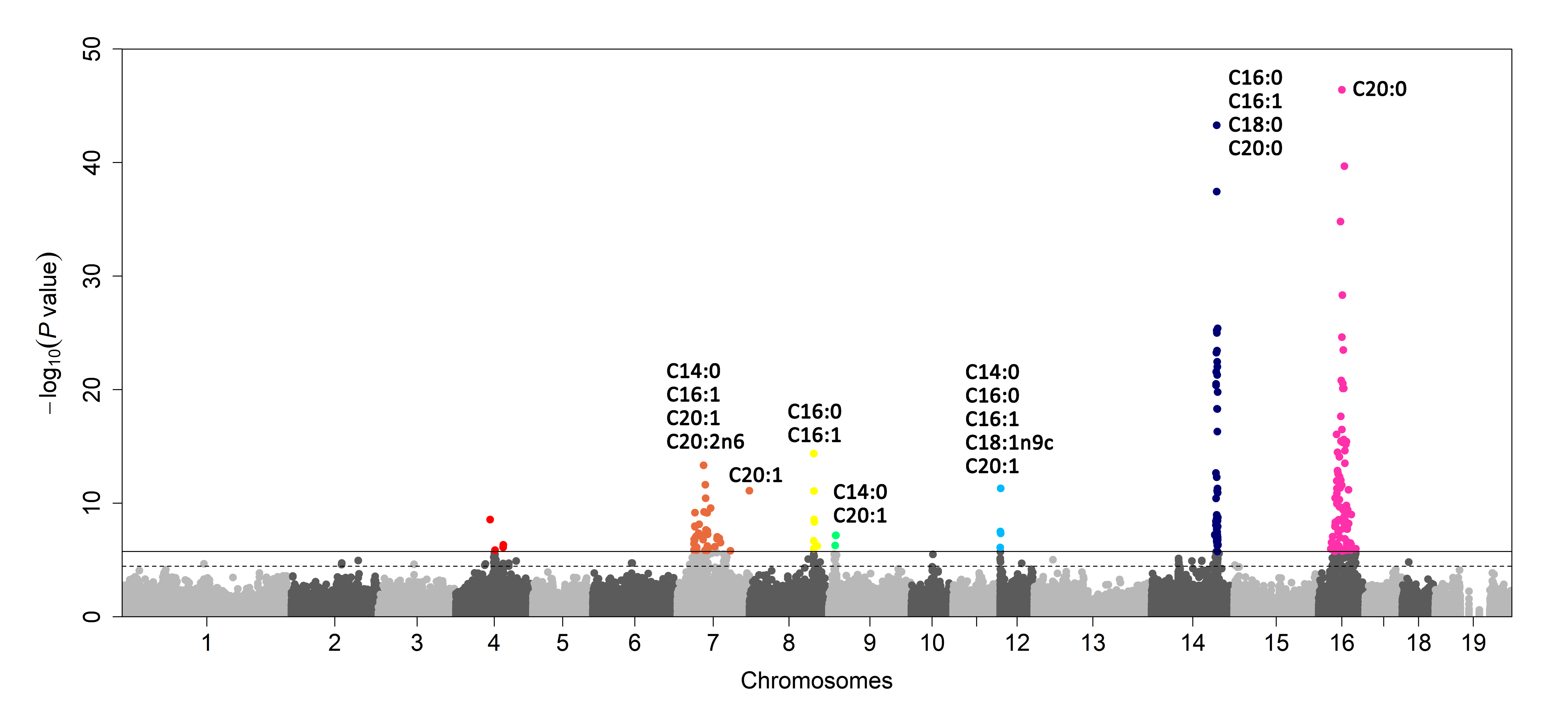

Supplement: Supplementary file 1 — 10.1186/s12711-016-0184-2 Manhattan plot for the GWAS meta-analysis for the content of 12 fatty acids in five populations, including Erhualian, Laiwu, DLY, Sutai and White Duroc × Erhualian F2 intercross. The solid line represents the genome-wide threshold (0.05/28025). The dashed line indicates the suggestive threshold (1/28025). Significant associations that surpass the genome-wide threshold are plotted in different colors on each chromosome. The fatty acid traits associated with the genome-wide significant loci are marked above the top SNPs. [file 12711_2016_184_MOESM1_ESM.tif]
