# Supplementary material for: Genetic architecture of fatty acid composition in the longissimusdorsi muscle revealed by genome-wide association studies on diverse pig populations
Source: Genet Sel Evol. 2016 Jan 21;48:5. doi: 10.1186/s12711-016-0184-2 (PMC4722735; doi:10.1186/s12711-016-0184-2)
Supplement: Supplementary file 2 — 10.1186/s12711-016-0184-2 Loci of suggestive significance identified by GWAS for fatty acid composition in Laiwu, Erhualian and DLY pig populations. [file 12711_2016_184_MOESM2_ESM.docx]

**Additional files 2: Table S1.** Loci of suggestive significance identified by GWAS for fatty acid compositions in Laiwu, Erhualian and DLY pig populations ^a^

| **Chr** | **Trait** | **Pop** | **Top SNP** | **Position (bp)** | ***P* value** | **Var (%)** | **MAF** |
| --- | --- | --- | --- | --- | --- | --- | --- |
| 4 | C14:0 | DLY | MARC0006929 | 86715104 | 6.34E-06 | 1.95 | 0.2098 |
| 7 | C14:0 | DLY | ALGA0110121 | 24797757 | 1.19E-05 | 1.62 | 0.06486 |
| 9 | C14:0 | DLY | ALGA0107040 | 13826057 | 2.15E-08 | 3.33 | 0.4926 |
| 9 | C14:0 | DLY | MARC0100725 | 14217867 | 3.23E-07 | 4.00 | 0.05082 |
| 9 | C14:0 | DLY | ALGA0103844 | 38447487 | 1.35E-05 | 3.05 | 0.4541 |
| 12 | C14:0 | DLY | ASGA0054450 | 40315806 | 8.10E-06 | 5.42 | 0.168 |
| 16 | C14:0 | DLY | ALGA0105383 | 55699815 | 9.38E-06 | 5.71 | 0.4351 |
| 23 | C14:0 | DLY | ALGA0100025 | 115829136 | 3.08E-06 | 2.11 | 0.3184 |
| 4 | C16:0 | DLY | ALGA0026434 | 94053848 | 8.61E-07 | 4.01 | 0.1861 |
| 14 | C16:0 | DLY | ALGA0081087 | 120972588 | 4.00E-08 | 6.25 | 0.2627 |
| 7 | C16:1n-7 | DLY | ASGA0035496 | 103286283 | 1.85E-05 | 1.44 | 0.01967 |
| 8 | C16:1n-7 | DLY | H3GA0025321 | 119887525 | 1.54E-13 | 8.31 | 0.0468 |
| 13 | C16:1n-7 | DLY | ASGA0104338 | 28524131 | 9.51E-06 | 2.83 | 0.2549 |
| 14 | C16:1n-7 | DLY | ASGA0066120 | 121515129 | 1.47E-13 | 11.13 | 0.327 |
| 15 | C16:1n-7 | DLY | ASGA0070271 | 117434181 | 1.05E-05 | 2.96 | 0.1362 |
| 13 | C18:0 | DLY | ALGA0124564 | 31252986 | 3.13E-06 | 3.76 | 0.3852 |
| 14 | C18:0 | DLY | MARC0063250 | 121500518 | 2.82E-25 | 26.35 | 0.3434 |
| 8 | C18:1n-9 | DLY | ALGA0046715 | 18115969 | 9.74E-06 | 5.57 | 0.4098 |
| 11 | C18:1n-9 | DLY | ASGA0051657 | 76165093 | 1.90E-05 | 5.55 | 0.2844 |
| 4 | C20:0 | DLY | ALGA0026631 | 97549734 | 1.46E-05 | 2.91 | 0.2885 |
| 14 | C20:0 | DLY | CASI0010164 | 121305916 | 1.04E-07 | 3.51 | 0.375 |
| 16 | C20:0 | DLY | DRGA0016155 | 43534471 | 4.32E-31 | 31.79 | 0.1189 |
| 7 | C20:1n-9 | DLY | ALGA0041580 | 53559770 | 2.19E-06 | 5.34 | 0.4557 |
| 9 | C20:1n-9 | DLY | ALGA0105252 | 6304559 | 4.27E-07 | 3.99 | 0.2992 |
| 16 | C20:1n-9 | DLY | DRGA0016155 | 43534471 | 8.98E-07 | 4.78 | 0.1189 |
| 2 | C20:3n-6 | DLY | ASGA0095320 | 8599190 | 1.15E-05 | 2.97 | 0.3432 |
| 2 | C20:3n-6 | DLY | ASGA0011178 | 113074904 | 2.90E-06 | 3.60 | 0.4268 |
| 4 | C20:3n-6 | DLY | ALGA0026905 | 102706847 | 3.88E-06 | 2.85 | 0.3262 |
| 1 | C20:3n6 | EHL | INRA0003300 | 101977893 | 2.21E-05 | 4.95 | 0.3601 |
| 4 | C14:0 | EHL | M1GA0005901 | 64622740 | 1.43E-06 | 11.03 | 0.1692 |
| 8 | C14:0 | EHL | ASGA0039925 | 134674491 | 1.51E-05 | 14.43 | 0.3452 |
| 9 | C14:0 | EHL | ASGA0084863 | 12366531 | 2.21E-05 | 8.09 | 0.1239 |
| 12 | C14:0 | EHL | MARC0063090 | 1779278 | 6.05E-12 | 26.81 | 0.1979 |
| 12 | C14:0 | EHL | M1GA0015780 | 2293399 | 1.78E-05 | 7.48 | 0.1178 |
| 12 | C14:0 | EHL | H3GA0055229 | 6882971 | 9.21E-06 | 2.20 | 0.297 |
| 12 | C14:0 | EHL | ALGA0067281 | 62407052 | 4.18E-06 | 7.37 | 0.3735 |
| 12 | C16:0 | EHL | MARC0063090 | 1779278 | 9.24E-09 | 19.56 | 0.1979 |
| 17 | C16:0 | EHL | ASGA0075696 | 22183010 | 1.64E-05 | 2.05 | 0.2872 |
| 7 | C16:1n-7 | EHL | ALGA0040052 | 33032424 | 7.11E-06 | 11.12 | 0.2507 |
| 8 | C16:1n-7 | EHL | ALGA0049349 | 127072110 | 6.18E-09 | 9.77 | 0.3943 |
| 12 | C16:1n-7 | EHL | MARC0063090 | 1779278 | 2.01E-10 | 13.07 | 0.1979 |
| 12 | C16:1n-7 | EHL | ASGA0052511 | 2621917 | 9.60E-06 | 0.33 | 0.189 |
| 12 | C18:0 | EHL | ASGA0103858 | 60801509 | 9.39E-06 | 7.28 | 0.4301 |
| 12 | C18:1n-9 | EHL | MARC0063090 | 1779278 | 1.14E-06 | 17.44 | 0.1979 |
| 12 | C18:1n-9 | EHL | H3GA0055229 | 6882971 | 2.63E-05 | 4.32 | 0.297 |
| 1 | C18:1n-9 | EHL | ASGA0004020 | 107964728 | 1.95E-05 | 2.48 | 0.3021 |
| 6 | C18:1n-9 | EHL | H3GA0018292 | 74354607 | 6.25E-06 | 7.80 | 0.4792 |
| 8 | C18:1n-9 | EHL | DIAS0001596 | 32155082 | 1.81E-06 | 7.71 | 0.01637 |
| 9 | C18:1n-9 | EHL | MARC0062322 | 145277018 | 7.33E-06 | 7.59 | 0.2642 |
| 13 | C18:1n-9 | EHL | DIAS0004475 | 75597972 | 1.66E-05 | 0.40 | 0.378 |
| 8 | C18:3n-3 | EHL | DIAS0001596 | 32155082 | 1.28E-07 | 9.11 | 0.01637 |
| 2 | C20:0 | EHL | ALGA0014301 | 89887451 | 2.97E-05 | 6.01 | 0.3657 |
| 3 | C20:0 | EHL | MARC0099803 | 127625936 | 7.33E-06 | 6.88 | 0.05357 |
| 7 | C20:0 | EHL | ALGA0044220 | 108116458 | 1.77E-05 | 14.78 | 0.1414 |
| 16 | C20:0 | EHL | ASGA0072949 | 34715842 | 6.97E-13 | 12.07 | 0.4762 |
| 16 | C20:0 | EHL | ASGA0073404 | 55993053 | 2.59E-05 | 2.95 | 0.02232 |
| 17 | C20:0 | EHL | ASGA0076365 | 35557609 | 1.21E-05 | 4.48 | 0.06832 |
| 4 | C20:1n-9 | EHL | MARC0031223 | 25283221 | 2.20E-06 | 4.11 | 0.04613 |
| 6 | C20:1n-9 | EHL | MARC0045581 | 9663840 | 2.36E-06 | 2.58 | 0.3403 |
| 7 | C20:1n-9 | EHL | DRGA0007276 | 19232432 | 4.96E-06 | 10.58 | 0.3408 |
| 7 | C20:1n-9 | EHL | INRA0027664 | 104914125 | 2.20E-05 | 9.99 | 0.3616 |
| 7 | C20:1n-9 | EHL | ALGA0114746 | 134540651 | 1.51E-17 | 26.43 | 0.2036 |
| 12 | C20:1n-9 | EHL | MARC0063090 | 1779278 | 1.03E-07 | 10.97 | 0.1979 |
| 14 | C20:1n-9 | EHL | DRGA0013964 | 68757221 | 2.41E-05 | 3.65 | 0.4643 |
| 1 | C20:2n-6 | EHL | H3GA0002769 | 145991718 | 2.04E-05 | 1.80 | 0.4881 |
| 3 | C20:2n-6 | EHL | ALGA0103673 | 42944414 | 3.02E-05 | 9.29 | 0.4953 |
| 5 | C20:2n-6 | EHL | H3GA0017242 | 103886357 | 1.65E-05 | 4.45 | 0.2024 |
| 6 | C20:2n-6 | EHL | ALGA0116528 | 72847504 | 1.14E-05 | 10.60 | 0.4747 |
| 7 | C20:2n-6 | EHL | ALGA0114746 | 134540651 | 9.81E-06 | 10.28 | 0.2036 |
| 8 | C20:2n-6 | EHL | DIAS0004322 | 138501500 | 5.87E-06 | 9.86 | 0.0403 |
| 16 | C20:2n-6 | EHL | ALGA0091575 | 74982852 | 1.97E-05 | 5.51 | 0.1414 |
| 2 | C20:3n-6 | EHL | MARC0111536 | 12089821 | 1.36E-05 | 1.30 | 0.2695 |
| 7 | C14:0 | Laiwu | ALGA0039950 | 31500144 | 2.60E-08 | 17.78 | 0.3592 |
| 12 | C14:0 | Laiwu | ASGA0099260 | 248014 | 4.06E-10 | 9.01 | 0.3987 |
| 7 | C16:0 | Laiwu | ASGA0085466 | 31380542 | 4.44E-06 | 11.58 | 0.2025 |
| 8 | C16:0 | Laiwu | ALGA0049254 | 120996107 | 2.66E-07 | 8.89 | 0.2073 |
| 6 | C16:1n-7 | Laiwu | ASGA0103013 | 4472594 | 1.36E-05 | 3.33 | 0.3259 |
| 6 | C16:1n-7 | Laiwu | ASGA0106005 | 71011913 | 1.97E-07 | 4.95 | 0.4747 |
| 7 | C16:1n-7 | Laiwu | DRGA0007448 | 31628039 | 1.27E-08 | 8.03 | 0.3813 |
| 9 | C16:1n-7 | Laiwu | ALGA0051583 | 14447589 | 5.63E-06 | 7.58 | 0.182 |
| 9 | C16:1n-7 | Laiwu | ALGA0054895 | 129654372 | 1.13E-05 | 3.11 | 0.1329 |
| 12 | C16:1n-7 | Laiwu | ALGA0123789 | 13486734 | 1.13E-05 | 13.63 | 0.4114 |
| 12 | C16:1n-7 | Laiwu | ALGA0067072 | 57831831 | 1.46E-05 | 13.04 | 0.2286 |
| 10 | C18:0 | Laiwu | MARC0047936 | 39007437 | 1.78E-05 | 3.37 | 0.3244 |
| 12 | C18:0 | Laiwu | MARC0093419 | 60329151 | 4.66E-06 | 11.50 | 0.2389 |
| 8 | C18:1n-9 | Laiwu | ALGA0049173 | 117981832 | 4.10E-06 | 6.63 | 0.2199 |
| 12 | C18:1n-9 | Laiwu | ALGA0111965 | 61495603 | 4.19E-06 | 6.23 | 0.4256 |
| 9 | C20:0 | Laiwu | ASGA0044318 | 122215309 | 4.68E-06 | 5.85 | 0.2769 |
| 16 | C20:0 | Laiwu | ASGA0073192 | 43402965 | 1.45E-06 | 2.65 | 0.3481 |
| 16 | C20:0 | Laiwu | DRGA0016169 | 45313348 | 7.41E-14 | 25.50 | 0.03165 |
| 7 | C20:1n-9 | Laiwu | H3GA0020505 | 29416373 | 2.25E-08 | 7.91 | 0.3465 |
| 7 | C20:1n-9 | Laiwu | ASGA0033712 | 53633798 | 1.26E-05 | 11.29 | 0.0981 |
| 7 | C20:1n-9 | Laiwu | ASGA0037322 | 133962789 | 3.54E-17 | 35.18 | 0.3117 |
| 12 | C20:1n-9 | Laiwu | ALGA0114537 | 13603120 | 1.45E-05 | 6.93 | 0.2516 |
| 12 | C20:1n-9 | Laiwu | ALGA0067173 | 60510869 | 7.09E-06 | 7.11 | 0.394 |
| 15 | C20:1n-9 | Laiwu | MARC0092224 | 146379361 | 4.08E-07 | 4.49 | 0.02373 |
| 7 | C20:2n-6 | Laiwu | DRGA0007448 | 31628039 | 5.61E-07 | 6.55 | 0.3813 |
| 12 | C20:2n-6 | Laiwu | MARC0004607 | 58950245 | 4.79E-06 | 6.97 | 0.3402 |
| 1 | C20:3n-6 | Laiwu | ASGA0005113 | 182196969 | 7.40E-06 | 7.18 | 0.1661 |
| 12 | C20:3n-6 | Laiwu | ASGA0055316 | 59580256 | 8.93E-06 | 5.50 | 0.4636 |
| 1 | C20:4n-6 | Laiwu | ASGA0004231 | 121663779 | 4.97E-06 | 10.16 | 0.01108 |
| 8 | C20:4n-6 | Laiwu | ALGA0049269 | 121743168 | 2.93E-06 | 4.26 | 0.4571 |

^a^ Chr, chromosome; Pop, population; *P*-values were calculated by the use of the GenABEL package in R. The phenotypic variation explained by the top SNPs was calculated by (V_reduce_ - V_full_)/V_resuce_, where V_reduce_ and V_full_ are residual variances of models with and without including SNP genotypes as predictor variables, respectively.
